# Silk-Derived 3D-Bioprinted Scaffolds for Neural Repair and Nerve Regeneration: A Comprehensive Review

**DOI:** 10.3390/life16060892

**Published:** 2026-05-26

**Authors:** Alynah J. Adams, Sanjana Challa, Cynthia Yan, Isabella Beltz, Alexa Kambol, Kaavian Shariati, Jocelyn Hunt, Charlotte Thomas, Dorien I. Schonebaum, Jose A. Foppiani, Umar Choudry, Samuel J. Lin

**Affiliations:** 1 Division of Plastic Surgery, Beth Israel Deaconess Medical Center, Harvard Medical School, Boston, MA 02215, USA; ajadams@mcw.edu (A.J.A.); sc6red@virginia.edu (S.C.);; 2 Medical College of Wisconsin, School of Medicine, Milwaukee, WI 53226, USA; 3 School of Medicine, University of North Carolina at Chapel Hill, Chapel Hill, NC 27599, USA; 4 Division of Plastic and Reconstructive Surgery, University of Minnesota, Minneapolis, MN 55455, USA; foppi002@umn.edu (J.A.F.); choud008@umn.edu (U.C.)

**Keywords:** nerve regeneration, neural repair, silk fibroin, silk bioink, 3D bioprinting, nerve guidance conduits, mechanical properties, tissue engineering

## Abstract

Traumatic injuries often result in nerve tissue damage and functional deficits due to limited regeneration. Silk fibroin, a biopolymer with inherent biocompatibility and tunable properties, is a promising material for 3D-bioprinted neural tissue scaffolds. This review highlights recent advancements in silk-derived composite scaffolds, often incorporating additional materials like collagen or conductive polymers to enhance their performance. This review examines how material composition, scaffold architecture, and fabrication strategy influence biological response and functional recovery. This comprehensive review follows PRISMA guidelines and uses comprehensive searches of PubMed, MEDLINE, Embase, Web of Science, Cochrane Central, and ClinicalTrials.gov for studies published through 2025. Studies were screened for eligibility based on substance type, mechanical properties, production methods, and outcomes. Findings were synthesized qualitatively. Twelve studies were included, comprising rat (50%), canine (8.3%), and in vitro (41.7%) models. Analysis reveals that silk fibroin acts as a highly adaptable mechanical backbone. It can consistently integrate with bioactive additives (collagen, dECM) or conductive polymers (Polypyrrole, MXene) to meet specific therapeutic demands. For spinal cord injuries, composites reached a compressive modulus capable of resisting physiological pressures and preventing scaffold collapse. In soft tissue applications, silk–hydrogel blends provided localized release of exosomes and small molecules during the acute injury phase, reducing neuroinflammatory markers. Additionally, adding conductive materials allowed the scaffolds to bridge electrical gaps and promote Schwann cell proliferation and neuronal differentiation. Furthermore, 3D bioprinting enabled the creation of defined microchannels that replicate native fascicular architecture. In vivo outcomes consistently showed superior axonal regeneration, myelination, and synaptic reconnection compared to controls, correlating with significant improvements in electrophysiological and motor function. This review highlights the clinical potential of silk fibroin-based 3D-printed biomaterials for nerve regeneration, including neural repair and neural tissue engineering. More recent studies place greater emphasis on integrating mechanical, architectural, and biological considerations into scaffold design, resulting in increasingly multifunctional scaffold systems. Despite promising efficacy, the heterogeneity of fabrication methods and the predominance of rodent models highlight the need for standardized protocols and evaluations in relevant models to facilitate clinical translation.

## 1. Introduction

Peripheral and central nerve injuries pose a significant challenge to individuals and the healthcare system, affecting millions of individuals annually. Injuries to nervous tissue from trauma, iatrogenic causes, or neurodegenerative disease frequently result in the permanent loss of motor, sensory, and autonomic function [1]. These deficits are associated with lifelong disability, chronic pain, and major reductions in quality of life [2]. A recent study found that individuals with peripheral nerve injuries reported significant impairment in quality of life [3]. The economic burden is also considerable, encompassing both direct medical costs, including surgery and rehabilitation, and indirect costs, including lost productivity and prolonged sick leave [4,5,6].

The current clinical gold standard for repairing large-gap peripheral nerve injuries is the autologous nerve graft. However, this approach is inherently limited by donor-site morbidity, sensory loss, neuroma formation, and finite donor tissue availability [7,8]. Furthermore, the limited availability of donor tissue, coupled with frequent mismatches in nerve diameter and fascicular architecture, contributes to inconsistent, often poor functional outcomes [9,10], with a 2022 review indicating that less than 50% of patients regain satisfactory function [11]. While acellular allografts and hollow synthetic conduits are also used clinically, they are generally appropriate for only small nerve gaps and have not consistently restored function across larger defects [12,13]. Together, these limitations underscore a persistent need for improved strategies for neural repair and nerve regeneration.

Three-dimensional (3D) bioprinting has emerged as a promising platform in tissue engineering to address this need. Unlike conventional fabrication methods, it enables precise, layer-by-layer deposition of bioinks to create high-resolution scaffolds that can be further tuned or fabricated according to patient-specific needs or constraints [14,15]. For nerve regeneration, scaffolds can be designed to mimic the native 3D architecture of a nerve and to provide the controlled spatial and temporal release of therapeutic cues [16]. The success of a 3D-bioprinted scaffold is influenced by the bioink. Silk fibroin (SF), a natural protein from *Bombyx mori* cocoons, is a particularly attractive biomaterial because of its biocompatibility, low immunogenicity, tunable mechanical properties, controllable biodegradability, and existing status as an FDA-approved material for medical devices [17,18]. In its native form, however, SF lacks the rapid gelation properties required for high-resolution extrusion bioprinting. This limitation has been overcome by chemical modification into derivatives, such as Silk Methacryloyl (SilMA), a photocurable polymer that crosslinks upon light exposure [19,20]. This modification transforms SF into a versatile bioink that can accurately print complex, stable 3D structures suitable for neural scaffolding.

The application of 3D-bioprinted silk-derived materials for nerve regeneration is a rapidly advancing field. Current preclinical studies focus on investigating novel silk-based composites, incorporating electrical conductivity into scaffolds, and printing constructs embedded with living cells [20,21,22]. While broad reviews on SF biomaterials, scaffold fabrication strategies, and 3D bioprinting approaches in tissue engineering exist, these topics are often discussed in isolation [18,23,24]. To date, no review has been published that synthesizes the evidence exclusively for 3D-bioprinted silk and SilMA scaffolds in nerve regeneration.

Therefore, the primary aim of this review is to identify and synthesize available preclinical studies that utilize 3D-bioprinted silk-derived scaffolds for neural repair. The goal is to provide an integrated analysis of 3D-bioprinted silk-derived scaffolds that emphasizes how material composition, scaffold architecture, and fabrication strategy collectively influence biological response and functional recovery. By isolating 3D-bioprinted applications of SF, we introduce an anatomical matching framework. The current literature frequently reviews silk fibroin purely as a biological substance but does not discuss how the mechanical realities of a given surgical defect may impact the success of SF scaffolds, particularly when applied in 3D-printed constructs. This framework highlights the importance of directly linking defect geometries to the required physical architecture of the printed scaffold. We provide a mechanical decision matrix that previous biochemical reviews on 3D printing of SF lack.

The synthesized data intend to clarify whether the current stage of 3D-printed silk-based scaffolds demonstrates enhanced outcomes compared to hollow conduits or non-printed alternatives. Further, the limitations and key challenges across the included studies will be examined to identify existing barriers to clinical translation and to guide discussion of future directions that may be pursued in this space.

## 2. Materials and Methods

### 2.1. Systematic Review

#### 2.1.1. Eligibility Criteria

Eligibility criteria included studies of 3D-printed silk derivatives for medical applications that addressed our primary and secondary outcomes, observational studies, and clinical trials published in English, French, or Spanish. Editorials, literature reviews, systematic reviews, meta-analyses, commentary reports, abstracts, and letters to the editor were excluded from this review because they typically do not present original research data. Due to this lack of originality, the required methodological rigor for inclusion could not be met. In addition, studies reporting treatments that did not 3D-print scaffolds or use silk derivatives, articles with no mechanical property data, and ongoing, pediatric, and cadaveric studies were excluded. Ongoing studies cannot provide convincing final results, and cadaveric studies have inherent limitations in translating findings to living subjects. The review protocol was submitted for registration with Prospero under ID 1238644.

#### 2.1.2. Information Sources

**Figure 1:**
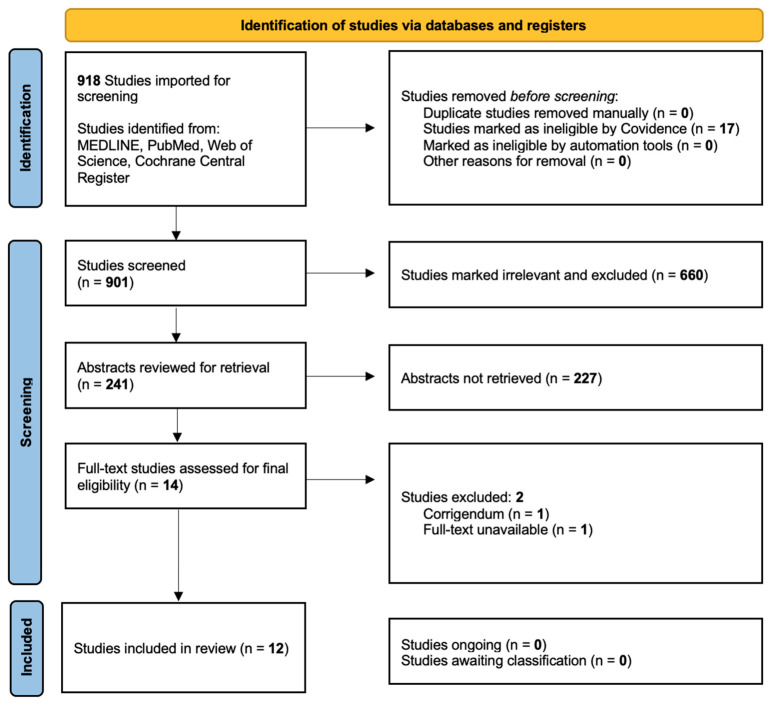
PRISMA flow diagram illustrating the identification, screening, eligibility assessment, and inclusion of studies in this review. Initial database queries utilizing predefined search strings returned an initial pool of candidate articles. Following abstract screening and the removal of duplicate records, researchers evaluated the remaining full-text manuscripts against strict inclusion criteria. Studies relying on conventional fabrication techniques were entirely excluded in an attempt to eliminate the confounding biological effects of random porosity. Ultimately, 12 preclinical animal studies focused exclusively on 3D-bioprinted silk composites met the final criteria for complete qualitative synthesis.

A comprehensive literature review was conducted in July 2025, following the Preferred Reporting Items for Systematic Reviews and Meta-Analyses (PRISMA) guidelines (Figure 1) [25,26]. The databases searched included PubMed, MEDLINE, Embase, Web of Science, Cochrane Central Register, and ClinicalTrials.gov, and 918 articles were imported into the systematic review screening platform, Covidence (Covidence systematic review software, Veritas Health Innovation, Melbourne, Australia) [27]. Ethical review and approval were waived for this study because the included studies did not involve human subjects.

#### 2.1.3. Search Strategy

The search strategy was designed by an experienced librarian using terms, subject headings, and keywords related to 3D printing, silk, biomaterial, bioengineering, biology, clinical outcomes, mechanical properties, physical properties, biomedical application, fibroin, wound dressing, tissue scaffolds, drug delivery, biocompatibility, biodegradability, medical therapy, and medical application. To investigate contemporary products and techniques for nerve repair, this review focused specifically on 3D-bioprinted scaffolds, excluding articles that did not focus on nerve repair. The complete search strings and Boolean operators utilized in this review are provided in Supplementary Table S1.

#### 2.1.4. Selection Process

The online systematic review screening program, Covidence, was used to evaluate the imported articles for review [27]. The automated Covidence platform pre-screened all articles, effectively removing 17 duplicate records based on overlapping bibliographic metadata, including title, author list, journal, publication year, and DOI, when available. Two independent reviewers conducted a two-stage screening process for study selection. First, records were identified through screening of article titles and abstracts. 901 records focused on 3D silk bioprinting remained. A post hoc decision was made to further focus the search on nerve regeneration only, removing 660 records for irrelevance (Figure 1), further highlighting the specific literature gap in 3D silk bioprinting for nerve regeneration. Of the remaining 241 records, 227 were not retrieved due to irrelevance, as the full-text did not fit the scope of this study despite the abstract initially seeming to. 14 articles were assessed for final eligibility. 2 additional articles were removed due to unavailability of full-text and corrigendum. Ultimately, 12 studies were included in a full-text analysis by the same two reviewers. All discrepancies were resolved by a third researcher, who moderated discussions until a joint decision was reached. The decision to limit the inclusion to 12 studies represents a deliberate methodological choice rather than a lack of available data. As described, hundreds of papers report on silk fibroin for neural applications. However, including conventional fabrication methods obscures the distinct advantages of additive manufacturing, which provides precise microarchitectural control. To accurately evaluate how specific internal geometries affect axonal guidance, we had to exclude any study that did not utilize true 3D bioprinting. This strict filtering isolates the exact variable under investigation. Consequently, the resulting dataset is small but highly specific.

#### 2.1.5. Data Extraction

Using predesignated variables, five independent reviewers extracted the data from the final articles. These variables included the first author’s last name, publication year, journal title, country of origin (China, Taiwan, or UK/China), study environment (in vivo, in vitro, or mixed), the model system utilized (rat, canine, or proof-of-concept in vitro models), the production method (low-temperature extrusion, 3D bioprinting, stereolithography, or electrospinning), the SF-based material or composite (e.g., SF alone, collagen/SF, SF/methacrylated gelatin (GelMA)/decellularized brain matrix (dECM), MXene-SilMA, PPy/SF, or yarn-hydrogel composites), and the primary and secondary outcomes.

#### 2.1.6. Data Items

The primary outcomes sought were axonal regeneration, neuroinflammatory markers, scaffold characterization, biocompatibility, functional recovery, synaptic integrity markers, degradation profile, and relevant molecular or immunologic endpoints. Other variables for which we sought data included author, title, year, publication year, journal, country of publication, and experimental approach.

#### 2.1.7. Statistical Analysis

The types of study outcomes included in this review varied considerably, precluding any analysis beyond a qualitative synthesis.

#### 2.1.8. CAMRADES Quality Assessment

To assess potential bias, the quality assessment checklist developed by the Collaborative Approach to Meta-Analysis and Review of Animal Data from Experimental Studies (CAMRADES) was used. The tool comprised 10 questions assessing potential bias in the study design. When a study achieved a ‘10’ on the rating scale, it indicated the strongest quality possible [28].

### 2.2. Literature Review

#### 2.2.1. Search Strategy

A comprehensive literature review was conducted in July 2025. The search strategy included PubMed, MEDLINE, Web of Science, Cochrane Central, and ClinicalTrials.gov, incorporating keywords, title headings, and abstracts for all papers published before this date.

#### 2.2.2. Study Selection

The search included editorials, systematic reviews, and original articles on 3D-printed silk-fibroin biomaterials for nerve regeneration. Studies were excluded if they focused only on electrospun materials not used in 3D printing, lacked silk in the biomaterials, did not improve nerve growth or regeneration, or did not clearly describe the mechanical properties, materials or processing methods. The objective was to highlight advancements in silk-based 3D bioprinting that offer clinical and translational benefits for patients.

#### 2.2.3. Data Extraction and Synthesis

A narrative synthesis of the included studies was conducted to examine the application of silk-derived 3D printing in nerve tissue regeneration.

## 3. Results

### 3.1. Study Selection and Evidence Characteristics

A total of 12 studies published between 2018 and 2025 were included in this review (Figure 1). The bibliographic characteristics, study environments, and specific silk fibroin-based material compositions, and quality score for each included article are summarized in Table 1.

Most studies were conducted in China (75%), with additional work from Taiwan (16.7%) and one collaborative UK/China study (8.3%) [29,30,31,32,33,34,35,36,37,38,39,40]. The majority were in vivo studies (66.7%), with 25% in vitro, and one mixed study (8.3%). Rat models were the most common (50%), followed by proof-of-concept studies (41.7%) and one canine model (8.3%) [29,30,31,32,33,34,35,36,37,38,39,40]. The included articles had CAMRADES quality scores ranging from 3 to 6 out of 10, with a median of 5. Three studies (25%) [33,36,39] scored 3 points; 2 studies (16.6%) [29,31] scored 4 points; 3 studies (25%) [30,32,40] scored 5 points; and 4 studies (33.3%) [34,35,37,38] scored 6 points, indicating that the overall methodological quality of the included studies was moderate (Table 1). These scores largely stem from a lack of reported blinding and randomization in the preclinical animal.

**Figure 2:**
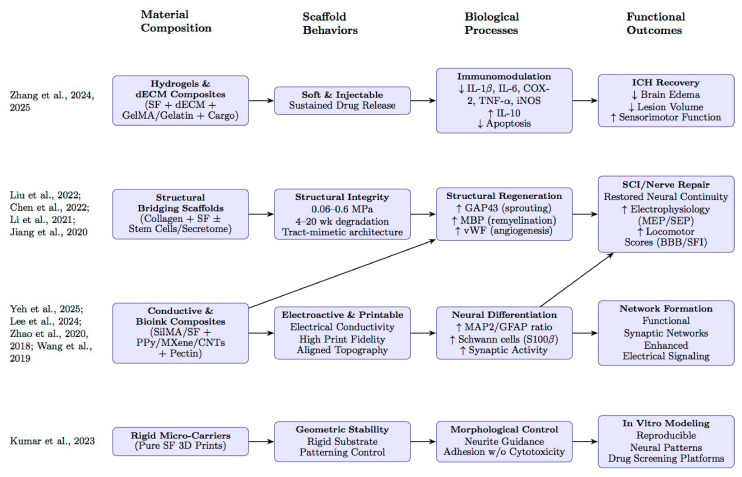
Three-dimensional biomaterial composition to functional outcomes in silk-based neural scaffolds. The diagram demonstrates how the initial three-dimensional biomaterial composition directly determines the scaffold’s physical behavior. Additive manufacturing enables precise control over scaffold behavior through targeted material blends. These engineered physical properties subsequently elicit specific biological responses at the injury site, such as targeted cell adhesion and directed axonal migration. These cellular interactions ultimately underpin the primary therapeutic mechanisms required for structural repair. The arrows trace how each material category progresses from scaffold composition to scaffold behavior, then to the biological processes observed in each study, and finally to the reported functional outcome; diagonal arrows indicate overlapping mechanisms where structural regeneration or neural differentiation contributes to broader repair outcomes across scaffold types [29,30,31,32,33,34,35,36,37,38,39,40].

The twelve included studies varied significantly in scaffold design and intended clinical application. Based on the data extraction, we categorized the studies into four primary material strategies: hydrogels, structural-bridging scaffolds, conductive bioinks, and microcarriers. Figure 2 provides an overview of these categories, including the physical properties of scaffolds used in each study and reported outcomes.

### 3.2. Analysis of Individual Studies and Clinical Applications

While each study is unique in its approach, capabilities, and clinical applicability, they all aimed to create a 3D bioink-printed silk-derived scaffold that promotes neurite growth. This section provides a broad overview of the studies included in this review before delving into their shared fabrication methods, functional outcomes, and mechanical properties (Table 2, Figure 2).

#### 3.2.1. Acute Intracranial Repair, Neuroprotection, and Localized Therapeutic Delivery

To treat intracerebral hemorrhage (ICH), Zhang et al. (2025) developed an injectable, 3D-printed scaffold composed of dECM, GelMA, and SF (Table 2 and Table 3) [29]. The scaffold carried and delivered human umbilical cord mesenchymal stem cell-derived exosomes and successfully sustained release for 28 days. This delivery effectively attenuated neuroinflammation and reduced neuronal apoptosis. The scaffold also improved neurological function in vivo as evidenced by function tests, and reduced brain edema relative to controls. However, the study also noted several important limitations: nanoparticle tracking analysis indicated that approximately 12% of exosomes entered the systemic circulation via the cerebrospinal fluid, raising the risk of off-target effects in peripheral organs. Therefore, further development of this technology to minimize systemic effects is needed prior to safe clinical translation. Further areas for development include compressing the scaffold to under 1 mm^3^ to enable minimally invasive neuroendoscopic implantation and minimizing batch-to-batch variability in exosome loading [29].

Similarly, in 2024, Zhang et al. engineered a photosensitive, injectable 3D biological scaffold combining dECM, SF, and GelMA to support ICH recovery (Table 2 and Table 3) [31]. The objective was achieved by delivering Bergenin, also known as arbutin. This natural compound, traditionally used in herbal medicine, exhibits anti-inflammatory, antioxidant, antibacterial, and antitumor properties. The scaffold released Bergenin continuously for three days, resulting in a significant reduction in neuroinflammation and oxidative stress. Furthermore, in vivo rat models showed that the treatment decreased hematoma volume, mitigated brain edema, and improved sensorimotor function compared with controls. The authors noted that the scaffold demonstrated excellent biocompatibility and, therefore, a low risk of rejection relative to cell therapies. Furthermore, they emphasize that the major clinical challenge this scaffold helps address is the rapid clearance of free drugs. Normally, a similar treatment would require repeated, invasive intracranial injections, but this scaffold would allow for prolonged local drug retention [31].

Liu et al. (2022) used a collagen and SF scaffold loaded with hypoxia-preconditioned mesenchymal stem cell exosomes to treat traumatic brain injury (TBI) in a canine model (Table 2 and Table 3) [34]. The scaffold was fabricated using low-temperature extrusion 3D printing. The study demonstrated that hypoxic preconditioning combined with SF significantly enhanced exosome efficacy, as evidenced by robust angiogenesis, inhibition of neuronal apoptosis, and a shift from a pro-inflammatory (IL-6, TNF-α) to an anti-inflammatory (IL-10) immune profile within the lesion. Furthermore, in vivo implantation promoted substantial nerve fiber regeneration and remyelination. This led to significant motor function recovery as quantified by modified Galasne, NDS, and Purdy scores. From a clinical standpoint, using exosomes in place of stem cells sidesteps issues such as tumor risk and immune rejection. Additionally, the large-animal model makes the findings more relevant to human treatment than traditional rodent studies [34].

#### 3.2.2. Structurally Persistent Bridging Scaffolds for Spinal Cord Repair

Li et al. (2021) used a 3D-printed collagen/silk fibroin scaffold to repair complete spinal cord transections in rats (Table 2 and Table 3) [36]. The scaffold contained microchannels arranged to imitate spinal white matter tracts. Compared to freeze-dried C/SF controls, the 3D-printed implants produced markedly better electrophysiological outcomes and locomotor recovery. On histological assessment, reduced cyst formation and more extensive axonal regeneration across the lesion were observed. Furthermore, the scaffold’s microchannel structure facilitated orderly axon extension and myelination across the glial scar barrier. This success in reconnecting proximal and distal spinal segments in an orderly fashion, despite structural obstacles posed by scar tissue, suggests that 3D printing could enable the reproduction of the complex microarchitecture of spinal white matter. However, this study has limited clinical relevance, as most SCIs are contusions rather than complete transections. The authors note that the scaffold should be tested in contusion models to improve its clinical applicability [36].

Jiang et al. (2020) employed low-temperature 3D bioprinting to engineer a scaffold composed of collagen and SF, with NSCs seeded therein (Table 2 and Table 3) [38]. This scaffold was used to bridge spinal cord transections in rats. The study found that the 3D-printed construct provided superior mechanical support compared with freeze-dried controls and fully resorbed within 4 weeks, being replaced by new neural tissue. In vivo application of the NSC-containing scaffold also showed improvements relative to controls, with decreased glial scar formation and improved axonal regeneration and remyelination. Furthermore, these structural improvements restored electrophysiological activity and improved motor outcomes. The study attributed the success of the SF/collagen scaffold to its tract-mimetic architecture, which enabled controlled axon guidance. However, the authors noted limitations, including the absence of NSC labeling, the need for more objective motor assessments, and the lack of corticospinal tract tracing; they suggest that future studies should address these gaps to better characterize the mechanisms of repair [38].

Chen et al. (2022) 3D-printed a collagen and SF scaffold functionalized with the secretome of human umbilical cord mesenchymal stem cells designed to bridge complete spinal cord transections in rats (Table 2 and Table 3) [35]. The scaffold successfully released 79 distinct therapeutic proteins, which, together, created a microenvironment that significantly enhanced NSC adhesion and proliferation in vitro compared with scaffolds lacking the secretome. In vivo, the construct successfully bridged the lesion and supported substantial axonal regrowth, remyelination, and synaptic restoration. These structural improvements were consequently accompanied by recovery of hindlimb locomotor function. The authors note that, because this secretome strategy is cell-free, it is safer and more controlled than conventional stem cell transplantation, potentially avoiding complications such as poor graft survival, immune rejection, and tumor formation. However, they note that further work is needed to elucidate which molecular mechanisms drive cell differentiation and oxidative stress reduction before this therapy can be used to manage human spinal cord injury (SCI) [35].

#### 3.2.3. Aligned and Electroactive Constructs for Peripheral Nerve Guidance

Zhao et al. (2018) aimed to promote peripheral nerve regeneration by electrospinning SF fibers coated with the conductive polymer PPy to create a conductive, nanofibrous scaffold (Table 2 and Table 3) [40]. The study found that the PPy coating imparted significant electrical conductivity (1.6 × 10^−2^ S/cm) to the scaffold while preserving the biocompatibility and advantageous mechanical properties of the SF core. The conductive PPy/SF scaffold also significantly enhanced cell adhesion, proliferation, and migration compared to pure SF scaffolds, as observed in the study’s in vitro experiments with Schwann cells. The construct also upregulated neurotrophic gene expression, such as S100β, NGF, and BDNF. The authors posit that this composite could serve as a bioactive alternative to autografts, as it could electrically stimulate nerve repair to bridge peripheral nerve gaps translationally. However, they caution that excess PPy content may lead to brittleness, potentially compromising the scaffold’s structural integrity during long-term implantation; therefore, the optimal PPy concentration must be carefully determined before clinical translation [40].

Wang et al. (2019) developed a 3D biomimetic core–shell scaffold to mimic the architecture of native peripheral nerve tissue (Table 2 and Table 3) [39]. The core was produced via dry-wet electrospinning and consisted of aligned conductive nanofiber yarns (NFYs) fabricated from polycaprolactone, SF, and carbon nanotubes. The shell was composed of photocrosslinked GelMA hydrogel. In vitro studies using PC12 cells and DRG explants showed that aligned conductive NFYs promoted greater neurite outgrowth and more directed migration than random substrates; neurites on 25 µm yarns extended up to ~1380 µm. The authors propose this core–shell design as a superior alternative to single-component conduits, as this design not only provides topographic guidance for axonal regrowth (via the core), but also a 3D hydrogel microenvironment (via the shell) that mimics the epineurium to protect regenerating tissue and potentially deliver neurotrophic factors such as nerve growth factor (NGF) or BDNF [39].

Zhao et al. (2020) used 3D-printed SF together with polypyrrole (PPy), a material that is easy to make and process, has good mechanical strength, and conducts electricity well, to create a nerve guidance conduit that helped repair peripheral nerve defects (Table 2 and Table 3) [37]. Their experiments found that applying electrical stimulation to this construct increased Schwann cell survival and migration and triggered the release of neurotrophic factors, including brain-derived neurotrophic factor (BDNF), NT-4/5, and NGF. The conduit was highly successful in rat models: it drove robust axonal regeneration and remyelination and achieved functional recovery comparable to that of an autograft. Although the experiment was highly successful, the authors point out that the cell-free design, the lack of built-in biochemical cues, and the simplicity of the injury model do not fully replicate real biological conditions. They suggest incorporating Schwann cells or stem cells into future experimental designs to better reflect the complexity of real clinical nerve injuries. The authors also noted that long-term in vivo stability and potential cytotoxicity arising from degradation should be investigated and addressed before the technology is recommended for translation to human studies [37].

#### 3.2.4. Cell-Laden and Patterned Biofabrication Platforms for Neural Maturation and In Vitro Modeling

Kumar et al. (2023) conducted a two-part study that produced porous poly(lactic-co-glycolic acid) (PLGA) spheres to support rapid fibroblast expansion and 3D-printed SF carriers to control neuronal patterning (Table 2 and Table 3) [33]. Our review focused on the neuronal aspect of the study. The SF carriers were shown to provide a chemically stable and geometrically flexible architecture: they supported PC12 neuronal adhesion and proliferation and facilitated directed neurite extension in the presence of nerve growth factor (NGF). The authors posit that the SF carriers are particularly suited for building patterned nerve grafts and for neurotrophic drug discovery. They also noted that moving the platform toward clinical application will require the development of more physiologically relevant co-culture models before use in a clinical setting [33].

Lee et al. (2024) set out to design a highly printable, mechanically tunable bioink for neural tissue engineering (Table 2 and Table 3) [32]. To achieve this, they combined photo-curable SilMA for structural stability and biocompatibility with ionic-crosslinking pectin to enhance the viscosity and shear-thinning properties of the bioink, thereby supporting highly detailed 3D bioprinting of NSC spheroids. Encapsulated NSCs maintained high viability (≥80–95%) and, as evidenced by a significant upregulation of Synapsin I, spontaneously differentiated into synaptically mature, complex neural networks with a high neuron: astrocyte ratio. The authors suggest that this platform could be integrated with chip technologies that have potential for clinical drug screening. They also note that the material is highly tunable, as SilMA and pectin can be varied independently, enabling precise adjustment of stiffness, printing fidelity, and other properties. Although pectin improves printability, excess pectin weakens the final structure. Therefore, a careful material balance should be determined before use in clinical contexts [32].

Yeh et al. (2025) developed a photo-crosslinkable, conductive bioink combining SilMA, pectin, and MXene-SP, designed specifically for 3D bioprinting of NSC spheroids (Table 2 and Table 3) [30]. The study found that MXene-SP showed significantly enhanced electrical conductivity and biocompatibility compared to unmodified MXene. Pectin improved the shear-thinning behavior necessary for printing delicate, high-resolution structures without damaging the NSCs. The spheroids that showed the greatest increase in NSC proliferation were exposed to daily electrical stimulation at 300 µA. This stimulation also induced NSCs to differentiate into MAP2+ neurons rather than astrocytes, which is beneficial for spinal cord function. Additionally, synaptic activity was also increased, as evidenced by FM1-43 dye uptake. However, the authors state that, before this bioink can be used translationally, future studies should optimize the scaffold’s porosity, stacking ability, and extensibility to ensure it maintains its shape, supports cell growth, and functions safely during movement and implantation in human patients [30].

### 3.3. Material and Fabrication Characteristics

To meet the inclusion criteria for this review, all studies incorporated an SF component to enhance mechanical, biochemical, or conductive properties, combined with other biomaterials that supplemented the properties of SF or served a different biological purpose (Table 1) [29,30,31,32,33,34,35,36,37,38,39,40]. Table 2, discussed below, details the functional roles of the biomaterials in each study.

Zhang et al. (2025) developed injectable dECM- and GelMA-based hydrogels to deliver exosomes, and Zhang et al. (2024) developed similar hydrogels to deliver Bergenin to modulate inflammation in intracerebral hemorrhage (ICH) models [29,31]. SF was used in these studies to provide soft, ECM-mimetic mechanical properties.

Kumar et al. (2023) and Wang et al. (2019) created scaffolds to guide neurite outgrowth and alignment [33,39]. Kumar et al. (2023) used SF to create a rigid, adhesive, structured, free-floating carrier to guide 3D growth of neurons [33], while Wang et al. (2019) used SF, PCL, and CNTs to create a nanofiber yarn to guide linear growth and a GelMA hydrogel shell to mimic epineurium [39].

Lastly, the three studies that focused on spinal cord injury (SCI) repair, Chen et al. (2022), Li et al. (2021), and Jiang et al. (2020), all incorporated collagen-SF structural composites into their scaffold [35,36,38]. Liu et al. (2022) applied a similar collagen-SF composite for traumatic brain injury (TBI) regeneration in canines [34]. These constructs were produced via low-temperature extrusion [34] or 3D printing [35,36,38] and were implanted intraoperatively. Each of these studies reported that collagen contributed to the ECM-like biological activity, and SF provided mechanical reinforcement.

### 3.4. Scaffold Delivery and Implantation (Table 2)

Delivery methods varied according to scaffold design and experimental model (Table 2). Injectable systems were selected in Zhang et al. (2024, 2025) to enable minimally invasive in vivo administration [29,31]. Intraoperative implantation was used for structurally rigid or semi-rigid 3D-printed collagen/SF scaffolds [34,35,36,38]. Hydrogel or encapsulation systems were used in Wang et al. (2019) and in the NSC spheroid bioprinting platforms created by Yeh et al. (2025) and Lee et al. (2024) [30,32,39]. Composite nerve conduits or conductive scaffolds were also used in peripheral nerve studies [37,40].

### 3.5. Functional Performance and Outcomes (Table 4 and Table 5)

All twelve studies included in this review successfully fabricated their intended SF-based constructs, including injectable hydrogels, 3D-printed collagen/SF scaffolds, conductive nerve conduits, and nanofiber-based composites. Fabrication fidelity was consistently high across printing, extrusion, and electrospinning methods [29,30,31,32,33,34,35,36,37,38,39,40].

Mechanical performance and physical characterization varied by composite design (Table 4). 3D-printed collagen/SF scaffolds designed for spinal cord repair demonstrated compressive moduli that spanned from 60 kPa to ~0.60 MPa [36,38]. Scaffolds that incorporated PPy or MXene demonstrated significantly enhanced electrical conductivity while maintaining the favorable mechanical properties of SF. Zhao et al. (2020) reported that PPy deposition improved the tensile strength of SF conduits, and Yeh et al. (2025) observed that soybean phospholipid-modified MXene (MXene-SP) incorporation reduced Young’s modulus, which is favorable for neuronal differentiation [30,37]. Hydrogel formulations, including SF-GelMA-dECM matrices [29] and Bergenin-loaded hydrogels [31], exhibited soft, ECM-like viscoelastic properties that accommodate the soft mechanical demands of CNS tissues. NSC-compatible bioinks displayed stable, solid-like properties, with G′ > G″, making them suitable for printing NSCs [30,32].

Degradation behavior was assessed in nine studies, with each behavior reflecting the biological role of the SF construct in the experiment (Table 4). 3D-printed collagen/SF scaffolds reported by Li et al. (2021) and Jiang et al. (2020) were designed to exhibit predictable, time-dependent mass loss, with complete degradation by four weeks [36,38]. Zhao et al. (2020, 2018) created a stable, conductive scaffold to promote peripheral nerve regeneration [37,40]. Zhao et al. (2018) reported that scaffolds retained 66.67% of their structure after 30 days, while Zhao et al. (2020) observed a mass loss of only ~8% over 4 weeks [37,40]. The canine TBI implants from Liu et al. (2022) quantified a 6-month in vivo degradation profile [34]. The study used a 1:12 collagen-to-SF ratio scaffold, as it degraded by 30% at 2 months and fully by 5 months, a suitable time frame for TBI repair [34]. Zhang et al. (2025) designed their scaffold to prolong the activity of hUCMSC-exos, which were released over a 14-day period [29]. The study reported ~47% loss of bioluminescence over days 2–14 as an indirect measure of scaffold degradation, indicating that the construct remained intact long enough to sustain exosome release [29]. Although Zhang et al. (2024) did not quantify the scaffold’s degradation profile, they found that the scaffold slowed the breakdown and diffusion of Bergenin and released the drug in a controlled manner for roughly 3 days [29,31]. Chen et al. (2022) also did not quantify degradation but reported that the scaffold exhibited favorable crystallinity, which conferred stability and enabled control of the degradation rate [35].

#### 3.5.1. Functional Outcomes (Table 5)

Functional assessments were performed across a spectrum of CNS and PNS models using behavioral, electrophysiological, imaging, and histological testing modalities (Table 5).

##### Central Nervous System Models

In ICH studies, SF-based hydrogels reduced neuroinflammation. Zhang et al. (2025) reported decreases of 48% and 52% in MMP-2 and MMP-9 levels, respectively [29]. Zhang et al. (2024) reported that the 3D SF scaffold carrying Bergenin reduced expression levels of iNOS, COX-2, IL-1β, TNF-α, and IL-6 while upregulating IL-10 [31]. Behavioral tests in both studies also improved: rats treated with the bioactive scaffold demonstrated superior sensorimotor function in forelimb placement and corner turn tests. Additionally, these rats showed significant improvements in neurological deficit scores; specifically, motor impairment over five days was reduced compared with controls [29,31].

In CNS injury models, as described in Table 5, 3D-printed collagen/SF scaffolds promoted neuroregeneration, as evidenced by improved axonal regrowth and enhanced myelination compared to controls [28,34,35,36]. Li et al. (2021) and Liu et al. (2022) also noted the upregulation of the growth cone marker GAP43, which correlates with axon regeneration [34,36]. Functional recovery of motor impairment was also fairly consistent in most studies. Rat spinal cord injury studies reported significant gains in the Basso–Beattie–Bresnahan (BBB) locomotor scores, demonstrating restored electrophysiological conduction as measured by MEP/SEP amplitude testing [35,36,38]. This demonstrates efficacy in both neurite proliferation and functional nerve recovery. Liu et al. (2022) observed quantifiable motor improvements in the beagle dog TBI model after consistent maturation of differentiated nerve fibers, axonal neurons, and astrocytes [34]. NDS and mGCS scores were consistent with Purdy scores, indicating substantial recovery in behavioral function and locomotion for over 7 days after scaffold implantation.

In the rat SCI studies, spinal cord structural recovery, as characterized by reduced cavity volume and tract coherence, was confirmed using MRI and DTI [35,36,38]. These structural restorations are tightly linked to improved locomotor function in rat models. Specifically, MRI showed marked axonal regeneration and connections between the proximal and distal SCI sites [36]. These results demonstrate a promising treatment for promoting tissue repair after SCI. Additionally, successful synaptic reconstruction was evidenced by the upregulation of PSD95 alongside presynaptic markers: synapsin (SYN) in the canine TBI model, and SYP expression in Chen et al. (2022) [34,35]. Remyelination and synaptic reconnection are anatomically essential for the recovery of neurological function after SCI [35].

##### Peripheral Nervous System Models

Zhao et al. (2020) and Zhao et al. (2018) both demonstrated enhanced Schwann cell proliferation (S100+, EdU+) [37,40]. Additionally, Zhao et al. (2020) reported increased organized myelin formation (TEM) and improved sciatic functional index (SFI) scores in vivo, indicating the scaffolds’ efficacy in sciatic nerve recovery after injury [37].

Studies by Wang et al. (2019) and Kumar et al. (2023) showed that SF-based composite scaffolds support robust neurite extension and development [33,39]. Wang et al. (2019) reported substantial neurite elongation and alignment in PC12 and DRG cells cultured on conductive yarn-hydrogel composites, with DRG explants exhibiting outgrowth exceeding ~1.3 mm along the nanofiber cores [39]. Although quantitative data were unavailable, Kumar et al. (2023) noted significant neurite growth and adhesion on 3D-printed carriers after staining, highlighting porous PLGA carriers and inkjet-printed SF carriers as effective options for neurite support growth [33]. Bioprinted NSC platforms also exhibited neuronal differentiation, as marked by increases in MAP2 [30] and Synapsin I [31] expression.

### 3.6. Temporal Dynamics of Regeneration

The studies indicate that the beneficial therapeutic effects of the silk scaffold on nerve regeneration occur in two distinct time frames. On an acute level, silk scaffolds played a critical role in neuroprotection by mitigating the secondary injury cascade of inflammation and oxidative stress within the first 5–14 days. In rat models of ICH, 3D-printed SF-containing scaffolds facilitated the sustained release of exosomes or small-molecule drugs such as Bergenin, resulting in a significant reduction in neuroinflammatory cytokines (IL-1β, TNF-α, IL-6) and astrogliosis (GFAP expression) [29,31]. SF’s minimal immunogenicity is critical in the acute setting to temper the initial inflammatory response. The localized, sustained delivery of therapeutic agents at the injury site mitigates systemic inflammation. It thus creates an environment conducive to tissue preservation in the immediate hours and days following trauma.

At a chronic level, silk scaffolds have been shown to play a significant role in axonal regeneration and functional recovery. Several studies have found that the standard time frame for observing true regeneration is approximately 8 weeks, as evidenced by axonal growth, remyelination, and synaptic connections in the corticospinal tract [35,36]. Unlike the acute neuroprotective window, this long-term window targets structural repair. Interventions in this phase are designed to act as substrates to promote, guide, and sustain the slower process of neural tissue reconstruction. For example, Liu et al. (2022) demonstrated that scaffolds implanted in a canine model maintained structural integrity for up to 6 months, degrading slowly enough to support long-term tissue reconstruction without causing chronic compression [34]. This distinction is critical for designing and timing therapeutic strategies to target effective interventions following nerve injuries.

### 3.7. Therapeutic Delivery

The success of these scaffolds in the acute phase is primarily attributed to their ability to provide sustained, localized release of therapeutics. This efficiency is due to silk’s porous architecture, stability, and biodegradable components. The porous structure enables uniform loading and cargo storage within the matrix. The protein structure of silk preserves the biological activity and structural integrity of the embedded biologics, protecting them from degradation caused by heat, light, or enzymatic activity in the physiological environment [41,42]. This ensures therapeutics remain viable until they reach the target site. Furthermore, silk is naturally biodegradable, enabling the sustained release of therapeutic agents over days to weeks. Unlike a simple bolus injection, which yields a high peak concentration that rapidly dissipates, leading to systemic exposure and often requiring frequent redosing [43,44,45,46,47]. The localized delivery of silk scaffolds maximizes treatment at the desired site, thereby minimizing off-target effects and enhancing overall therapeutic efficacy.

### 3.8. Conductivity

**Figure 3:**
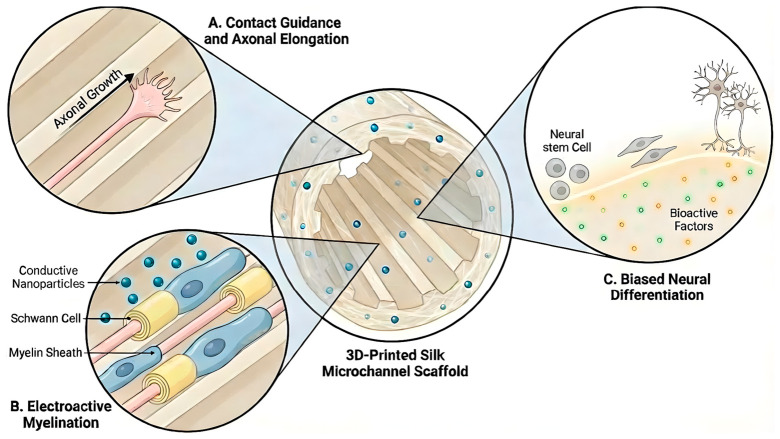
Cellular mechanisms modulated by 3D-printed composite silk fibroin scaffolds. 3D-printing and material blends influence cellular behaviors at the injury site. (**A**) 3D-printed microchannels provide physical contact guidance for organized axonal elongation, limiting disorganized neuroma formation. (**B**) The addition of conductive additives promotes Schwann cell myelination along the newly formed tracts. (**C**) Functionalized bioinks release local factors that bias neural stem cells toward mature neuronal lineages. These combined mechanisms demonstrate how structural and electrical tuning accelerates functional recovery compared to passive autograft models. Figure created using FigureLabs (Sora).

Beyond drug delivery, the studies reviewed provide compelling evidence for the critical role of electrical conductivity in neural repair. The novel composite materials, specifically the PPy/SF and MXene/SF Methacrylate (MXene/SilMA) systems, demonstrated that, when combined with electrical stimulation, silk scaffolds significantly promoted NSC differentiation and enhanced synaptic function. Zhao et al. (2020) demonstrated that PPy/SF composites created a conductive microenvironment that significantly enhanced Schwann cell proliferation (S100β+) and improved the g-ratio of regenerated myelin sheaths [37]. Similarly, Yeh et al. (2025) showed that conductive MXene-based bioinks upregulated neuronal differentiation markers in NSCs [30]. These advanced composites create an electroactive environment that mimics the electrical characteristics of native neural tissue. This environment facilitates enhanced electrical signal transmission, which is critical for neuronal differentiation, network formation, and maturation (Figure 3).

### 3.9. Architectural Guidance

Critically, these studies demonstrate that the 3D-printed scaffold structure could be important for guiding axonal regeneration. This finding highlights the importance of the structural design as more than passive support; it actively directs neuronal growth. The study by Kumar et al. (2023) confirmed that 3D-printed silk fibrin provides architectural guidance, as the model demonstrated patterned cell growth and neurite spreading in a directed 3D manner [33]. Li et al. (2021) and Jiang et al. (2020) designed silk scaffolds to mimic the structure of the corticospinal tract and achieved orderly axonal regeneration in vivo [36,38]. Similarly, the study by Wang et al. (2019) aligned nanofiber yarns within a silk scaffold to promote the alignment of regenerating nerve cells, as evidenced by neurite elongation as assessed by NF staining [39]. Beyond structural guidance, the studies indicate that these composite scaffolds actively sustain the continuum of neuronal development. Studies tracking NSCs observed a successful transition from early progenitors (Nestin+) into mature neurons (MAP2+) [30,32]. The eventual expression of synaptic markers, such as Synapsin [32] and PSD95 [35], suggests that these scaffolds foster not only the anatomical regrowth of neurons but also the re-establishment of communicating neural networks.

All studies demonstrated significantly improved functional recovery, suggesting that replication of native tissue architecture is essential for restoring neurological function. The 3D-printed silk scaffolds provide highly organized features to ensure nerve fibers regenerate along the correct axis and prevent the misdirected growth often seen in random-pore scaffolds [36,48]. This feature is crucial for bridging substantial gaps and ensuring that nerves accurately reinnervate their intended targets, representing a significant improvement over prior limitations [49,50,51,52,53]. In essence, these findings confirm that successful nerve regeneration strategies must move beyond simple biocompatibility and incorporate architectural guidance and structural mimicry to ensure functional connectivity is restored (Figure 3).

### 3.10. Design Synthesis and Strategies

The collective synthesis of the included studies, despite substantial heterogeneity in scaffold architecture, fabrication strategy, and intended clinical application, suggests that silk is more appropriately understood as a platform biomaterial than as a single scaffold class. In this context, the role of silk fibroin is not fixed, but instead varies according to the biological demands of the repair setting and the design features with which it is paired, including matrix composition, architecture, mechanical behavior, degradation profile, and adjunctive bioactivity. More broadly, this interpretation is consistent with the wider biomaterial literature, which has emphasized the versatility of silk fibroin across multiple tissue-engineering formats and the importance of tailoring scaffold properties to the target neural microenvironment [54,55].

Across the studies included in this review, four recurring design archetypes emerge (Table 6). First, silk-based systems can serve as soft, injectable, matrix-mimetic depots for acute intracranial repair, with the principal goals of local therapeutic retention, neuroprotection, and modulation of the secondary injury milieu, as illustrated by the intracerebral hemorrhage and brain injury studies by Zhang et al. and Liu et al. [31,34]. Second, silk can be configured into structurally persistent, architecturally defined bridges for spinal cord lesions, where channelization, tissue integration, and longer-term support of axonal regeneration and remyelination become the dominant design priorities [35,36,38]. Third, in peripheral nerve repair, silk is most often incorporated into aligned and, in some cases, conductive guidance constructs, in which internal organization and electroactive functionality are leveraged to support Schwann-cell behavior, neurite alignment, and restoration of conduction across a defect [37,39,40]. Finally, several studies position silk-derived bioinks as cell-laden or patterned platforms for neural maturation and in vitro modeling, emphasizing print fidelity, cytocompatibility, differentiation, and network formation rather than direct in vivo repair [30,32,33].

Notably, comparison across publication years suggests a temporal shift in scaffold design strategies. Earlier studies (2018–2022) primarily focused on structural integrity and mechanical support using SF in combination with collagen or synthetic polymers [34,36,37,38,39,40]. In contrast, more recent studies (2023–2025) place emphasis on bioactive and multifunctional scaffold systems. The newer constructs incorporate features such as sustained therapeutic delivery [29,31], electrical conductivity [30], and improved biofabrication approaches [32,33] to more actively modulate the regenerative environment.

**Figure 4:**
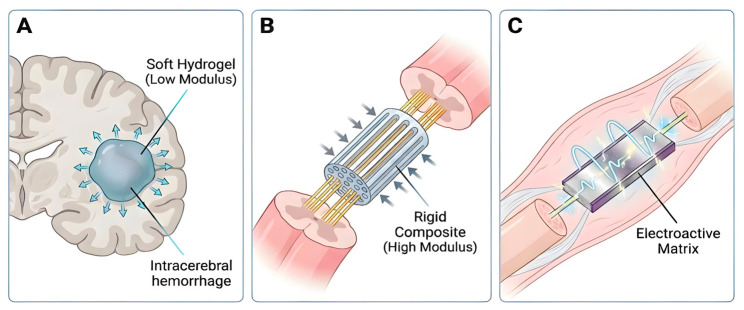
Biomaterial design strategies for 3D-printed silk scaffolds based on the anatomical defect. The physical specificities of the neural defect help identify the required material architecture. (**A**) Contained cavities require soft hydrogels that match the low compressive modulus of native brain tissue to facilitate localized molecular diffusion from the hydrogel to the tissue. (**B**) Physical gaps demand rigid, micro-channeled composites to resist physiological compression and provide structural guidance for axonal elongation. (**C**) Electrically inactive lesions require the integration of electroactive matrices to restore signal propagation across the defect space. Matching the biomaterial profile to the exact anatomical demands prevents secondary trauma and determines the success of neural repair. Figure created using FigureLabs (Sora).

Taken together, these patterns suggest that the translational relevance of silk fibroin lies less in any single intrinsic property than in its capacity to serve as a tunable structural and biologic backbone that can be adapted to distinct neural repair problems. This interpretation supports a conceptual framework in which scaffold performance is determined by the interaction between composition, scaffold architecture, and the biological environment, as reflected in the recent increased emphasis on multifunctional scaffold design. Framed in this way, the reviewed literature supports a use-case-specific view of scaffold design, in which the success of a silk-derived construct depends on how effectively its formulation and architecture are matched to the mechanical, spatial, and regenerative requirements of the target lesion rather than on the use of silk fibroin alone (Table 6) (Figure 4) [54,55,56,57,58]. Our previous work suggests that the micro- and macrostructural features of 3D-printed materials can influence the clinical integration of biomaterials for neural regeneration, particularly when designed to mimic the epineurial layer and thereby enhance translational potential [59]. Future epineurium-focused scaffold strategies may be further strengthened by leveraging the intrinsic anti-inflammatory properties of silk to better modulate the local injury microenvironment and support regenerative healing.

## 4. Discussion

This review demonstrates the ongoing development of 3D-printed silk-based scaffolds to address complex neural injuries, including SCI, TBI, and ICH, as well as peripheral nerve injury. The analysis of 12 studies published between 2018 and 2025 reveals a technological shift from passive conduits to bioactive, composite systems. The studies demonstrated that 3D-printed silk-based scaffolds are versatile, biocompatible materials that, when combined with bioactive additives, promote neurite outgrowth and facilitate functional recovery [29,30,31,32,33,34,35,36,37,38,39,40]. These outcomes are driven by silk scaffolds’ ability to deliver bioactive molecules, serve as a conductive interface to facilitate neural signaling, and guide tissue regeneration through architectural control.

### 4.1. Advantages of Silk Fibroin Compared to Other Biomaterials

The evolution of nerve repair strategies has been driven by the clinical necessity to bridge large, unrepairable nerve defects. The autologous nerve graft has long served as the clinical gold standard, utilized for its native Schwann cell population and basal lamina architecture [60,61]. However, the dependence on donor tissue introduced significant morbidity, including sensory loss at the harvest site and neuroma formation [9,10,62,63]. These limitations necessitated the development of synthetic alternatives.

First-generation nerve guidance conduits were simple, hollow tubes composed of non-resorbable silicone or polytetrafluoroethylene (PTFE). While effective for small gaps, these conduits frequently failed in larger defects due to the lack of internal architectural support, leading to fibrous scar infiltration rather than directed axonal regrowth [64,65]. Second-generation conduits utilized biodegradable synthetic polymers, such as polyglycolic acid (PGA) and polylactic acid (PLA). While these materials eliminated the need for surgical removal, they were often limited by acidic degradation byproducts that induced local inflammation, negatively affecting the delicate regenerative microenvironment [66,67]. The limitations of these second-generation conduits highlighted the need for a scaffold material that is not only biodegradable but also chemically inert during the resorption process. 

The 12 studies reviewed indicate that 3D-bioprinted silk fibroin composites offer functional advantages over these previous generations of biomaterials. Derived from *Bombyx mori* cocoons, SF is a natural protein polymer characterized by distinct hydrophobic beta-sheet crystallites that confer exceptional mechanical strength [68]. Unlike pure collagen or soft hydrogels, which lacks structural integrity and degrades rapidly, SF provides a robust mechanical background [17,18,69]. Other recent literature confirms this structural advantage as Dan et al. demonstrated that these beta-sheet structures directly prevent scaffold collapse under physiological loads, while Zhai et al. proved that cross-linking SF massively increases its compressive modulus, creating a stable mechanical backbone for tissue engineering [24,70].

Another key advantage is that SF avoids the acidic degradation byproducts like those associated with PLGA, by exhibiting a slow, tunable degradation profile that produces non-toxic amino acids [17,18,33,34,36,37,38]. These findings align with recent literature as several studies also found that the enzymatic degradation process of silk fibroin does not trigger cellular toxicity [24,71]. Instead, SF provides a stable environment for tissue repair that is crucial for neural microenvironments.

### 4.2. Advantages of 3D Bioprinting as a Fabrication Technique

An advancement highlighted by this review is the shift from random porosity to architectural control. Early fabrication methods included solvent coating [72], freeze-drying [73], and electrospinning [74,75,76], all of which produce materials with random porosity [77,78,79]. While beneficial for nutrient exchange [80], random pore structures fail to provide the directional guidance cues necessary for axonal pathfinding, often leading to disorganized nerve growth [48,81,82].

Comparisons with recent non-printed scaffold studies clarify the value of additive manufacturing. The use of 3D-bioprinting allows for the layer-by-layer deposition of bioinks to create specific geometries with precise internal microarchitectures [67,68]. This enables the creation of multichannel conduits that mimic the fascicular architecture of peripheral nerves or the tracts of the spinal cord [14,15,16]. As evidenced by the studies utilizing linear microchannels to mimic the fascicular architecture of peripheral nerves or spinal tracts, this precision helps guide axonal elongation to repair gaps (Figure 3) [35,36,37,38,39,40]. Additionally, these findings demonstrate that the structural alignment supports directed axonal growth and limits the disorganized neuroma formation that was previously observed using other biomaterials [35,36,38], which is in alignment with findings from recent reviews [24,71]. By providing physical support to regenerating tissue, 3D-printed SF provides a clear benefit by preventing disorganized growth that may have caused earlier synthetic conduits to fail.

### 4.3. Assessment of the Composite Scaffold Types

The rationale for categorizing these scaffolds is based on their specific biomechanical functions. We grouped the materials into hydrogels, structural scaffolds, conductive bioinks, and microcarriers because each class drives a distinct mechanism of neural repair. Hydrogels function primarily through localized diffusion. Structural scaffolds provide rigid, directional contact guidance to physically bridge severed axons in peripheral gaps. Conductive bioinks actively restore electrical signal propagation across inactive lesions to accelerate myelination. Finally, microcarriers act as delivery vehicles to protect seeded stem cells from immune clearance while maintaining targeted localization at the injury site. This mechanistic grouping provides a clear framework for understanding how physical material properties dictate biological recovery.

Following this framework, a central finding of this review is that SF can be mechanically and electrically tailored to the specific anatomical defect through multi-material composites. These findings are consistent with recent literature as several studies demonstrated that SF is not as effective in isolation. For example, Zhu et al. established that a single biomaterial cannot fully replicate the properties of complex tissues, requiring SF to be blended with other agents to address specific defect conditions [71]. Zhai et al. demonstrated this practically by incorporating SF into classic bioinks like GelMA. They found that this combination significantly improved both the printing resolution and the ultimate mechanical properties of the hydrogel [70]. Ultimately, the success of these material blends relies entirely on matching the composite architecture to the mechanical needs of the target defect.

Central nervous system repair presents different biological barriers compared to peripheral injuries. Spinal cord and traumatic intracranial injuries generate a highly inhibitory microenvironment where dense glial scarring and severe secondary neuroinflammation prevent regeneration. Thus, modulation of the host environment is needed to promote functional recovery. For acute intracranial injuries, soft hydrogels excel in these contained environments. Zhang et al. (2024) and Zhang et al. (2025) formulated dECM and SF blends with a low compressive modulus that matches native brain tissue [29,31]. This scaffold type prevents secondary mechanical trauma while permitting the highly efficient diffusion of delivered neurotrophic factors. However, these soft scaffolds do not function as well in gap-bridging applications. Without the structural rigidity to withstand spinal column compression and cerebrospinal fluid flow, soft hydrogels collapse in these environments.

On the other hand, peripheral nerve repair primarily presents a structural challenge. Clinical success relies on guiding Schwann cell migration across a physical gap to restore nerve conduction. Rigid composite scaffolds are better suited to withstand this mechanical pressure. By incorporating materials like collagen and using 3D-printed microchannels, Li et al. (2021) engineered a 3D-printed collagen/SF composite with a compressive modulus of approximately 0.60 MPa to allow the porous space to remain open [36]. Similarly, Liu et al. (2022) utilized this SF-reinforced structural integrity to ensure that scaffolds remained intact for up to 6 months in a canine model, while Chen et al. (2022) demonstrated scaffold stability over an 8-week observation period in a rat model [34,35]. By resisting degradation and mechanical compression, these rigid scaffolds are suited to provide stable tract for regenerating nerve fibers to traverse and repair defects [38].

However, only relying on structural support to repair nerve gaps can result in slow functional recovery. Conductive scaffolds are facilitative of this as they can integrate materials to actively modulate the local microenvironment and restore signal propagation. For example, Zhao et al. (2020) demonstrated that electroactive polypyrrole matrices promote Schwann cell myelination in peripheral nerves, and Yeh et al. (2025) found that MXene-based bioinks bias uncommitted neural stem cells toward mature neuronal lineages [30,37]. Current literature echoes these findings as incorporating additional functional materials has been shown to accelerate the establishment of synaptic connections compared to simple freeze-dried alternatives [24]. However, high concentrations of conductive polymers make scaffolds brittle and may result in loss of structural integrity [40].

Ultimately, each scaffold type plays a different biological role depending on the anatomical context (Figure 4). This mechanical tunability creates a permissive physical environment that can be utilized to address the specific pathological barriers presented by each distinct type of neural injury.

### 4.4. Innovations and Future Direction

The fundamental scalability of 3D-bioprinted silk remains a challenge in exploring its clinical applications. Standardizing Good Manufacturing Practice protocols for naturally derived proteins is difficult as batch-to-batch variability directly limits reproducibility. Natural silk cocoons contain inherent structural variations and unpredictable levels of impurities, requiring purification steps that may compromise the structure and biocompatibility of the final product [83]. Without universally standardized extraction methods, it is difficult to guarantee the exact mechanical properties of the bioink. Scaling this process for commercial 3D printing therefore invites regulatory rejection.

Due to these issues in scalability of 3D bioprinting, several alternative methods are being investigated for mass-producing SF scaffolds, including electrospinning, freeze-drying, freeze-thawing, and multilayer fabrication. Electrospinning is typically used to create nanofibrous SF structures, and is often combined with additional materials to enhance electrical conductivity, which is crucial for neural tissue repair [76,84,85]. Freeze-drying and freeze-thawing are processes that yield highly porous, bioresorbable SF conduits without the use of potentially harmful chemicals [55,86]. The multi-layered architecture is unique in that it is a multimodal approach in its own right, combining the aforementioned production methods [87].

As researchers refine fabrication techniques, Four-dimensional printing of SF is emerging. 4D printing of SF focuses on developing biocompatible, shape-morphing scaffolds to support tissue regeneration. Using digital light processing and photocurable SF hydrogels to create constructs capable of shape changes under physiologic conditions, tissue regeneration has been achieved [55,88]. However, long-term in vivo studies are still required to better understand how these structures interact with tissue.

Further work is being conducted to investigate other functional materials for multi-material SF 3D printing. As seen in several studies included in this review and others, SF can be blended with different materials such as collagen [34,38], pectin [32], sodium alginate, or polylactic acid [89], among others, to not only improve scaffold strength but also improve elasticity and porosity to more closely mimic native neuronal tissue, support cell survival, and enhance axonal guidance [90]. Additionally, incorporating bioactive molecules such as growth factors or stem cell secretomes [34], peptides [91], or functionalizing with nanoparticles [92,93] enhances cell adhesion, proliferation, differentiation, and neuroprotection. Lastly, multi-material scaffolds can be engineered for sustained release of growth factors or extracellular vesicles, supporting long-term tissue repair and modulating the injury microenvironment [34,94,95]. However, this introduces a significant barrier as composite biologics that combine scaffolds with living cells or active secretomes face steep FDA regulatory hurdles compared to passive synthetic conduits [96]. Combining SF with additional materials enhances neuronal repair and regeneration, but researchers must account for these complex regulatory pathways when considering further investigation into the optimal material combination.

### 4.5. Limitations

One major limitation is the restricted scope of the evidence base. Only 12 studies met the inclusion criteria. While the broader field of silk biomaterials contains numerous publications, isolating 3D-bioprinted scaffolds restricts the analysis to a specific technique that is not yet widely studied. Conclusions about efficacy are therefore preliminary and rely on emerging literature.

Another limitation across the reviewed literature is the heavy reliance on rodent models. Thus, drawing direct clinical comparisons is difficult. Small experimental sample sizes within these animal studies further weaken the predictive power of the findings. Success in a rodent model serves only as a basic proof of concept and does not guarantee clinical efficacy.

The quality of this review is limited to the quality of the included studies. While the studies successfully demonstrated structural regeneration, the median CAMRADES score remained a five out of ten. Most publications lost points because researchers failed to clearly report appropriate randomization or blinded outcome assessments. Moderate quality scores are consistently present in the broader field of preclinical tissue engineering. Researchers often prioritize complex material fabrication over rigorous animal trial design. This may introduce a risk of observer bias. Without strict blinding during functional testing, evaluators might unintentionally inflate the recovery scores of treated animals. This observer bias is a potential limitation of the findings of this review. If the original researchers exaggerated the functional outcomes, the perceived clinical advantage of additive manufacturing might be an artifact of poor study design rather than a true biological effect. Until independent laboratories replicate these results using fully blinded protocols, the biological superiority of these printed structures discussed in this review should remain a preliminary hypothesis rather than an established clinical outcome. Future preclinical trials must adopt rigorous blinding protocols to verify that observed improvements in nerve conduction and locomotion are objective.

An additional limitation is the brief observation period of these preclinical investigations. Most included studies evaluate functional recovery and scaffold degradation over weeks. Short-term animal models cannot accurately capture the long-term foreign body response, delayed immune rejection, or the complete degradation profile of the bioprinted silk composites. Without longitudinal data, establishing the true translational safety of these materials remains challenging. Future investigations should evaluate these scaffolds over extended, multi-year periods. These studies should determine if early functional gains are permanent and if scaffolds continue to be safe over a longer time period without inducing late-stage toxicity.

All reviews are also limited by publication bias, whereby studies with positive or statistically significant findings are more likely to be published and therefore included in the review. This review may be limited by outdated evidence, as reviews capture evidence only up to the last search date. Variability in search strategies and inclusion criteria can introduce selection bias and opportunities for reviewer bias, thereby increasing inter-rater variability despite the use of standardized protocols.

### 4.6. Challenges in Clinical Translation

Despite the promising applications of SF in regenerative medicine, several important limitations must be acknowledged when evaluating the suitability of this platform for large-scale clinical translation. True mechanical and rheological characterization is seldom described in the current literature. When considering the mass production of silk and its feasibility at large scales, batch variability remains a challenge in SF production, affecting its mechanical properties and the biocompatibility of scaffolds. Additionally, the structural variability and impurities of natural silk sources further challenge consistent scaffold performance at a large scale [55,58,97]. Currently, there is a lack of standardized extraction and processing methods for SF, leading to inconsistent material properties and reproducibility issues in clinical applications such as nerve regeneration [55,97,98]. The extraction of SF is complex and time-consuming, and may involve toxic chemicals, which can undermine the biocompatibility and clinical scalability [99,100,101]. SF solutions often require additives or blending with additional polymers to achieve suitable strength and maintain scaffold shape during and after printing, owing to silk’s rheological properties. This remains a technical hurdle to the large-scale reproducibility of materials [58,97,98,102]. Further investigation into the degradation rates and immune response requires extensive preclinical and clinical validation; however, the lack of standardized manufacturing processes complicates regulatory approval for clinical use.

## 5. Conclusions

This review suggests that 3D-bioprinted silk fibroin scaffolds can support neural regeneration in animal models. When reinforced with bioactive or conductive additives, these structures can provide architectural guidance, immunomodulation, and mechanical support. By synthesizing data on 3D-printed silk derivatives, this study clarifies how fabrication-dependent structural mimicry influences neural repair. These findings suggest that successful neural regeneration may require a multi-material approach to overcome the mechanical and biological limitations of traditional autografts or simple hydrogels. Ultimately, while these scaffolds demonstrate translational potential, clinical applications of this technology remain a distant target. Future success relies on standardizing fabrication protocols and demonstrating consistent efficacy in more clinically relevant models.

## Figures and Tables

**Table 1:** Article bibliometrics, study model, scaffold composition, and CAMRADES score rating.

Article Title	Authors, Year	Country of Origin	Study Model	Scaffold Composition	CAMRADES * Score (Out of 10)
Effects of 3D-printed exosome-functionalized brain acellular matrix hydrogel on neuroinflammation in rats following cerebral hemorrhage	Zhang, 2025 [29]	China	Rat	dECM + GelMA + SF + MSC exosomes	4
Innovative MXene/SilMA-Based Conductive Bioink for Three Dimensional Bioprinting of Neural Stem Cell Spheroids in Neural Tissue Engineering	Yeh, 2025 [30]	Taiwan	in vitro	SilMA + Pectin + MXene-SP + SF	5
Three-dimensional biological scaffold delivers Bergenin to reduce neuroinflammation in rats with cerebral hemorrhage	Zhang, 2024 [31]	China	Rat in vitro	dECM + Gelatin + SF + Bergenin	4
Dual crosslinking silk fibroin/pectin-based bioink development and the application on neural stem/progenitor cells spheroid laden 3D bioprinting	Lee, 2024 [32]	Taiwan	in vitro	SilMA + Pectin	5
Three-dimensional culture of fibroblasts and neuronal cells on microfabricated free-floating carriers	Kumar, 2023 [33]	UK China	in vitro	PLGA + SF	3
Hypoxia-pretreated mesenchymal stem cell-derived exosomes-loaded low-temperature extrusion 3D-printed implants for neural regeneration after traumatic brain injury in canines	Liu, 2022 [34]	China	Canine in vitro	Collagen + Hypoxia exosomes + SF	6
3D-printed collagen/silk fibroin scaffolds carrying the secretome of human umbilical mesenchymal stem cells ameliorated neurological dysfunction after spinal cord injury in rats	Chen, 2022 [35]	China	Rat in vivo	Collagen + SF + MSC secretome	6
The corticospinal tract structure of collagen/silk fibroin scaffold implants using 3D printing promotes functional recovery after complete spinal cord transection in rats	Li, 2021 [36]	China	Rat	Collagen + SF	3
Application of conductive PPy/SF composite scaffold and electrical stimulation for neural tissue engineering	Zhao, 2020 [37]	China	Rat in vitro	PPY + SF	6
Three-dimensional bioprinting collagen/silk fibroin scaffold combined with neural stem cells promotes nerve regeneration after spinal cord injury	Jiang, 2020 [38]	China	Rat in vitro	Collagen + NSCs + SF	6
Aligned conductive core–shell biomimetic scaffolds based on nanofiber yarns/hydrogel for enhanced 3D neurite outgrowth alignment and elongation	Wang, 2019 [39]	China	in vitro	PCL + SF + CNTs + GelMA	3
Novel conductive polypyrrole/silk fibroin scaffold for neural tissue repair	Zhao, 2018 [40]	China	in vitro	PPY + SF	5

SF: Silk Fibroin; PCL: Polycaprolactone; dECM: Decellularized extra-cellular matrix; GelMA: Gelatin methacryloyl; MSC: Mesenchymal Stem Cell; SilMA: Silk fibroin modified with glycidyl methacrylate; MXene-SP: Soybean phospholipid-modified titanium carbide; PPy: Polypyrrole; NSC: Neural Stem Cells; CNT: Carbon Nanotube * CAMRADES score is out of 10 points.

**Table 2:** Biological parameters, neuronal cell type targets, and neuronal markers.

Authors, Year	Role of Materials	Delivery Method	Neuronal Cell Type Targeted	Neuronal Markers
Zhang, 2025 [29]	dECM: biochemical cues; GelMA: photocuring; SF: structure; Exosomes: anti-inflammatory cargo	Injectable	Astrocytes; in vivo neurons	GFAP
Yeh, 2025 [30]	SilMA: matrix; Pectin: printability; MXene: conductivity	Hydrogel encapsulation	NSCs	Nestin, MAP2, GFAP, FM1-43
Zhang, 2024 [31]	SF: mechanical stability; dECM: biochemical cues; Gelatin: viscosity; Bergenin: anti-inflammatory	Injectable	Primary astrocytes/glia (in vitro); neurons and glia (in vivo)	GFAP
Lee, 2024 [32]	SilMA: UV-crosslinking; Pectin: ionic gelation + viscosity	Encapsulation	Neural stem/progenitor cells	Nestin, MAP2, GFAP, Synapsin
Kumar, 2023 [33]	SF: rigid, adhesive, structured 3D carrier	Seeding	L929, PC12	phalloidin/DAPI and SEM
Liu, 2022 [34]	Collagen: ECM bioactivity; SF: stability; Exos: neuroregenerative cargo	Intraoperative	NSCs (in vitro); cortical axons (in vivo)	Nestin, MAP2, Tuj1, NF, MBP, GAP43, PSD95, SYN, GFAP
Chen, 2022 [35]	Collagen: bioactivity; SF: mechanical stability; Secretome: neurotrophic cocktail	Intraoperative	NSCs (in vitro); Regenerating axons (in vivo)	NF, MBP, PSD95, SYP, Bielschowsky silver, TEM
Li, 2021 [36]	SF: mechanical strength; Collagen: ECM adhesion	Intraoperative	Spinal axons	NF, MBP, GAP43, BDA tracing
Zhao, 2020 [37]	SF: aligned fibers and stability; PPy: conductivity	Intraoperative	Schwann cells (in vitro and in vivo)	NF-H, S100, TEM myelin metrics
Jiang, 2020 [38]	Collagen: ECM bioactivity; SF: strength and stability; NSCs: regenerative cell therapy	Intraoperative	NSCs; spinal axons	Nestin, MAP2, βIII-tubulin, NF-H, GFAP
Wang, 2019 [39]	SF: biocompatibility and fiber formation; CNTs: conductivity; PCL: mechanical strength; Hydrogel shell: mimics epineurium	Encapsulation	PC12, DRG cells and explants	NF
Zhao, 2018 [40]	SF: stability and adhesion; PPy: conductivity	Seeding	Schwann cells	S100β, EdU

SF: silk fibroin; PCL: polycaprolactone; dECM: Decellularized extra-cellular matrix; GelMA: Gelatin methacryloyl; SilMA: Silk fibroin modified with glycidyl methacrylate; PPy: Polypyrrole; NSC: Neural Stem Cells; GFAP: Glial fibrillary acidic protein; MAP2: Microtubule-associated protein 2; Tuj1: βIII-tubulin; NF: Neurofilament; SEM: Scanning electron microscopy; MBP: Myelin basic protein; GAP43: Growth Associated Protein 43; TEM: Transmission electron microscopy; BDA: Biotinylated dextran amine; NF-H: Neurofilament heavy chain; CNT: Carbon Nanotube; DRG: Dorsal root ganglion; SYN: Synaptophysin.

**Table 3:** Silk 3DP instruments, approach, and settings.

Authors, Year	3D Printer	Manufacturer (Company, City Country)	Printing Approach	Printing Speed	Printer Height	Printer Needle Diameter	Scaffold Architecture
Zhang, 2025 [29]	Bio-Architect-WS	Regenovo Biotechnology, Ltd., Hangzhou, China	Extrusion-Based Bioprinting	6 mm/s	1 mm, unspecified layer height	340 μm	dECM + GelMA + SF
Yeh, 2025 [30]	N/A	Custom built, Taiwan,	Extrusion-Based 3D printing	1–30 mm/s	0–500 μm	413 μm	0.7 g GMA per gram SF; 2 × 2 stacked grid patterns
Zhang, 2024 [31]	Bio-Architect-WS	Regenovo Biotechnology, Ltd., Hangzhou, China	Extrusion-Based Bioprinting	6 mm/s	3 mm, unspecified layer height	100 μm	dECM + GelMA + SF + photoinitiator, with and without Bergenin loading
Lee, 2024 [32]	TL-D5 TMC2209	Custom built, China	Extrusion-based 3D bioprinting	1–30 mm/s	0–500 μm	413 μm	SilMA + Pectin + Photoinitiator with and without PecMA/SF system
Kumar, 2023 [33]	Piezo inkjet printer	Custom built, UK	Reactive inkjet printing	N/A	100–200 layers	80 μm	PLGA; SF
Liu, 2022 [34]	3D-Bioplotter™ system	Regenovo Biotechnology, Ltd., Hangzhou, China	Low-temperature extrusion printing	12 mm/s	2 mm, 0.3 mm per layer	160 μm	Collagen and SF (1:12 ratio)
Chen, 2022 [35]	3D-Bioprinter	Regenovo Biotechnology, Ltd., Hangzhou, China	Low-temperature extrusion printing	12 mm/s	3 mm, 0.3 mm per layer	160 μm	Collagen and SF (1:2 ratio) adsorbed with HUCMSC secretom
Li, 2021 [36]	3D-Bioprinter	Regenovo Biotechnology, Ltd., Hangzhou, China	Low-temperature extrusion printing	9 mm/s	3 mm, 0.1 mm per layer	210 μm	Collagen and SF (1:1 ratio)
Zhao, 2020 [37]	3D-Bioprinter	Regenovo Biotechnology, Ltd., Hangzhou, China	Low-temperature extrusion printing	Adjusted to nozzle scan speed	1.1 mm diameter, 10 mm length, unspecified layer height	260 μm	PPy and SF film with silk fibroin nanofiber shell
Jiang, 2020 [38]	3D-Bioprinter	Regenovo Biotechnology, Ltd., Hangzhou, China	Low-temperature extrusion printing	9 mm/s	2 mm, 0.1 mm per layer	210 μm	Collagen and SF (4:2 ratio)
Wang, 2019 [39]	Custom-built electrospinning apparatus	Custom built, China	Dry-wet electrospinning and photocrosslinking	70, 100, 120 mm/min	350–700 μm (nanofiber yarn diameters)	21 G (514 μm)	PCL + SF + CNTs (aligned nanofiber yarns); GelMA (hydrogel shell)
Zhao, 2018 [40]	3D-Bioprinter	Regenovo Biotechnology, Ltd., Hangzhou, China	Low-temperature extrusion printing	Adjusted to nozzle scan speed	80, 120, or 180 μm filament diameter, unspecified layer height	260 μm	PPy, SF, and SF nanofiber coating

**Table 4:** Mechanical and biological properties.

Authors, Year	Tensile Strength	Young’s Modulus (MPa)	Conductive Ability	Compressive Modulus (kPa)	Durability	Degradation Profile (Weeks)
Zhang, 2025 [29]	Not quantified	Not quantified	Not quantified	Not quantified	Not quantified	Not quantified
Yeh, 2025 [30]	Not quantified	Not quantified	Not assessed	~1–4 kPa	MXene/SilMA/pectin hydrogels durable over days of culture and printing handling, but long term durability was not assessed	Not quantified
Zhang, 2024 [31]	Not assessed	Not assessed	Not assessed	Not assessed	Not quantified	Not quantified beyond a 5 day observation period
Lee, 2024 [32]	Not assessed	Not assessed	Not assessed	Qualitative assessment	Higher SilMA and PecMA/SF concentrations and longer UV exposure was more robust, Pectin improved printability and shape maintenance	Not quantified
Kumar, 2023 [33]	Not assessed	Not assessed	Not assessed	Not assessed	Mechanically rigid; chemically stable	Biodegradable, timeline not assessed
Liu, 2022 [34]	Not assessed	Not assessed	Not assessed	Not assessed	Favorable physical properties; high water absorption	30% at 8 weeks, completely degraded at 22 weeks
Chen, 2022 [35]	Not assessed	1.69 ± 0.18 kPa 1.17 ± 0.31 kPa	Not assessed	35–40 kPa	SF provided mechanical strength, elasticity, and stability to compensate for collagen	Not quantified; defined at stable
Li, 2021 [36]	Not assessed	0.60 ± 12 MPa	Not assessed	Not quantified	High mechanical strength	20% degraded at 1 week, completely degraded at 4 weeks
Zhao, 2020 [37]	0.059 MPa	Not assessed	0.11446 ± 0.00145 mS/mm	Not assessed	Structure remained intact after demolding; smooth conduit surface	7.72% mass loss at 4 weeks
Jiang, 2020 [38]	Not assessed	Not assessed	Not assessed	~60.05 ± 5.12 kPa	Stable properties; provided space for NSC survival	Gradual degradation over 1–4 weeks; complete degradation by week 4
Wang, 2019 [39]	Not assessed	Not assessed	Not assessed	Not assessed	Three-dimensional alignment maintained during 7-day culture period	Not quantified
Zhao, 2018 [40]	Not assessed	Not assessed	1.82 ± 0.21 × 10^−5^ S/cm to 1.13 ± 0.19 × 10^−3^ S/cm	Not assessed	66.67% stability at 30 days	Not quantified

**Table 5:** Functional assessment, effectiveness of the Silk 3D biomimetic product, and complications.

Authors, Year	Function Assessment	Effectiveness of Silk 3D Biomimetic	Complications of Product
Zhang, 2025 [29]	Release kinetics, neurobehavioral tests in ICH rats, and histology and biomarkers	Improved neurobehavioral function, reduced brain edema and barrier leak, decreased inflammation and apoptosis	Systemic exposure of exomes, scaffold size for minimally invasive delivery, incomplete mechanistic understanding
Yeh, 2025 [30]	Maintenance of viable neuronal stem cells, migration toward neurons, and synaptic activity enhancement	Kept neuronal stem cells alive, amplified their growth, biased them toward neurons, and enhanced synaptic activity	Formulation and printing instability at higher MXene loadings; overly strong electrical stimulation can harm cells rather than overt toxicity of the biomimetic
Zhang, 2024 [31]	Bergenin delivery and release, anti-inflammatory and antioxidant effects. Cytoprotective and anti-apoptotic effects, and neurorepair and functional recovery	Scaffold loaded with Bergenin is more effective than free Bergenin or hydrogel carriers	Short observation window without assessment of long-term effects on brain repair past 5 days, potential for systemic toxicity, and required stereotactic in situ transplantation
Lee, 2024 [32]	Structural and mechanical behavior, printability, and ability to support cell survival, neural differentiation, and 3D network formation	SilMA/pectin prints reliably, remains structurally stable, is highly biocompatible, and strongly promotes neuronal differentiation and network formation	Narrow optimal formulation and processing range due to limitations in gelatin window, softness versus printability trade-offs, and UV exposure-dependent cytotoxicity
Kumar, 2023 [33]	Characterization of PLGA and fibroin carriers	Facilitated differentiation and acted as carrier for cell patterning and directed growth	Background fluorescence noise; significant cell sloughing during washing; large neuronal cells clusters underwent necrosis
Liu, 2022 [34]	Evaluate cell compatibility and nerve growth, connections, and function	Inhibited apoptosis and neuroinflammation; promoted neuroregeneration, axonal regrowth, and functional motor recovery	Challenges of direct stem cell implantation
Chen, 2022 [35]	Investigate repair of neural networks after spinal cord injury	Enhanced nerve regeneration, remyelination, and axonal conduction to improve motor function	Did not investigate if 3D-C/S + ST scaffold can promote cell differentiation, remove ROS, or reduce inflammation
Li, 2021 [36]	Guide axonal growth and repair spin cord by simulating corticospinal tract structure	Promoted axonal regrowth, myelination, and improved motor function	Did not assess scaffolds with nerve cells or growth factors
Zhao, 2020 [37]	Schwann cell proliferation/migration assays, Sciatic Function Index, TEM myelin metrics	Promoted axonal regeneration and remyelination in vivo	Excess electrical stimulation increased Schwann cell apoptosis/necrosis
Jiang, 2020 [38]	Behavioral scoring (BBB, inclined plane), Motor Evoked Potential, MRI/DTI for spinal continuity, histological analysis of injury cavity filling	Promoted nerve regeneration, reduced glial scarring, significantly improved motor function recovery, particularly when combined with NSCs	Does not isolate scaffold vs. NSC effect, lacks mechanistic investigation of repair pathways
Wang, 2019 [39]	In vitro viability and 3D alignment assessment of PC12 and DRG cells, measurement of neurite migration distance/alignment from DRG explants	Guided 3D neurite alignment and elongation with high directional accuracy	No in vivo repair, primarily structural/topographical validation
Zhao, 2018 [40]	Conductivity and 30-day stability tests in water/DMEM, MTT cytotoxicity, EdU proliferation, and S100β staining for Schwann cell arrangement	Supported Schwann cell growth and alignment	PPy hydrophobicity limited cell adhesion

3D-C/S + ST: secretomes derived from human umbilical mesenchymal stem cells, BBB: Basso-Beattie-Bresnahan open-field locomotor scoring, NSC: Neural Stem Cell.

**Table 6:** Scaffold Design Strategies and Functional Priorities by Regenerative Context.

Regenerative Context	Dominant Therapeutic Objective	Scaffold Design/ Precedent	Functional Priorities	Outcome Domains	Author, Year
Acute Intracranial Injury (ICH, Focal TBI Cavity)	Local neuroprotection, modulation of secondary injury, and sustained therapeutic delivery	Soft, injectable, matrix-mimetic hydrogel	Conformability to irregular cavities; low inflammatory burden; controlled release kinetics; compatibility with intracranial delivery; preservation of local tissue architecture	Neuroinflammatory markers, apoptosis/cell survival, edema/cavity evolution, neurologic and behavioral recovery	Zhang, 2025 [29]; Zhang, 2024 [31]; Liu, 2022 [34]
Spinal Cord Defect Repair	Structural bridging of tissue loss and support of organized axonal regeneration	Implantable, architecturally defined scaffold with persistent structural integrity	Channelized or tract-mimetic architecture; mechanical stability sufficient for bridging; host integration; support for axonal extension, remyelination, and synaptic reorganization; degradation matched to repair timescale	Axonal regeneration, myelination, synaptic marker expression, MRI/DTI continuity, electrophysiology, locomotor recovery	Chen, 2022 [35]; Li, 2021 [36]; Jiang, 2020 [38]
Peripheral Nerve Defect Repair	Directed axonal extension across a nerve gap and restoration of conduction	Guidance conduit or aligned fibrous/core–shell construct, with optional conductive functionality	Internal alignment cues; lumen patency and flexibility; Schwann-cell compatibility; suturability/handling; where used, conductivity integrated without compromising biocompatibility	Schwann-cell migration/proliferation, neurite alignment, myelination, nerve conduction, muscle reinnervation, functional index recovery	Zhao, 2020 [37]; Wang, 2019 [39]; Zhao, 2018 [40]
Cell-Laden Neural Biofabrication Platforms and In Vitro Neural Modeling	Support of cell viability, differentiation, maturation, and network formation within a printable 3D environment	Soft, high-fidelity cell-compatible bioink with tunable rheology and optional electroactivity	Print fidelity; cell survival during and after printing; diffusion/perfusion characteristics; support of neuronal differentiation and synaptic maturation; reproducibility across constructs	Viability, differentiation markers, neurite extension, synaptic activity, stimulation responsiveness, construct stability	Yeh, 2025 [30]; Lee, 2024 [32]; Kumar, 2023 [33]

## Data Availability

The original contributions presented in this study are included in the article/supplementary material. Further inquiries can be directed to the corresponding author.

## Supplementary Materials

The following supporting information can be downloaded at https://www.mdpi.com/article/10.3390/life16060892/s1. Table S1. Databases and search string utilized in study selection.

## Abbreviations

The following abbreviations are used in this manuscript:

3D	Three-dimensional
SF	Silk fibroin
FDA	Food and Drug Administration
SilMA	Silk methacryloyl
PRISMA	Preferred Reporting Items for Systematic Reviews and Meta-Analyses
GelMA	Gelatin methacryloyl
dECM	Decellularized extracellular matrix
PPy	Polypyrrole
CAMRADES	Collaborative Approach to Meta-Analysis and Review of Animal Data from Experimental Studies
MSC	Mesenchymal stem cell
MXene-SP	Soybean phospholipid-modified titanium carbide
PLGA	Poly(lactic-co-glycolic acid)
PCL	Polycaprolactone
NSC	Neural stem cell
CNT	Carbon nanotube
GFAP	Glial fibrillary acidic protein
MAP2	Microtubule-associated protein 2
FM1-43	FM1-43 fluorescent styryl dye
UV	Ultraviolet
DAPI	4′,6-diamidino-2-phenylindole
SEM	Scanning electron microscopy
Tuj1	βIII-tubulin
NF	Neurofilament
MBP	Myelin basic protein
GAP43	Growth-associated protein 43
PSD95	Postsynaptic density protein 95
SYN	Synapsin
SYP	Synaptophysin
TEM	Transmission electron microscopy
BDA	Biotinylated dextran amine
NF-H	Neurofilament heavy chain
DRG	Dorsal root ganglion
S100β	S100 calcium-binding protein β
EdU	5-ethynyl-2′-deoxyuridine
ICH	Intracerebral hemorrhage
TBI	Traumatic brain injury
IL-6	Interleukin 6
TNF-α	Tumor necrosis factor alpha
IL-10	Interleukin 10
NDS	Neurological Deficit Score
SCI	Spinal cord injury
NGF	Nerve growth factor
BDNF	Brain-derived neurotrophic factor
NFYs	Nanofiber yarns
NT-4/5	Neurotrophin-4/5
GMA	Glycidyl methacrylate
PecMA	Pectin methacrylate
HUCMSC	Human umbilical cord mesenchymal stem cell
CNS	Central nervous system
hUCMSC-exos	Human umbilical cord mesenchymal stem cell-derived exosomes
3D-C/S + ST	Three-dimensional collagen/silk fibroin scaffold with secretome
ROS	Reactive oxygen species
BBB	Basso-Beattie-Bresnahan open-field locomotor score
MRI	Magnetic resonance imaging
DTI	Diffusion tensor imaging
DMEM	Dulbecco’s Modified Eagle Medium
MTT	3-(4,5-dimethylthiazol-2-yl)-2,5-diphenyltetrazolium bromide
PNS	Peripheral nervous system
MMP-2	Matrix metalloproteinase 2
MMP-9	Matrix metalloproteinase 9
iNOS	Inducible nitric oxide synthase
COX-2	Cyclooxygenase-2
MEP	Motor evoked potential
SEP	Somatosensory evoked potential
mGCS	Modified Glasgow Coma Scale
SFI	Sciatic Function Index
PTFE	Polytetrafluoroethylene
PGA	Polyglycolic acid
PLA	Polylactic acid
4D	Four-dimensional
