# Supplementary material for: Silk-Derived 3D-Bioprinted Scaffolds for Neural Repair and Nerve Regeneration: A Comprehensive Review
Source: Life (Basel). 2026 May 26;16(6):892. doi: 10.3390/life16060892 (PMC13302405; doi:10.3390/life16060892)
Supplement: Supplementary file 1 [file life-16-00892-s001.zip › life-4342319-supplementary.pdf]

Table S1. Databases and search string utilized in study selection.

Name of the search database (screened via Covidence)	Search String
PubMed, MEDLINE, Embase, Web of Science, Cochrane Central Register, and ClinicalTrials.gov	"3D bioprinting" OR bioprinting OR "three-dimensional printing" OR biofabrication) AND (silk OR "silk fibroin" OR sericin OR "silk-based biomaterial" OR "silk hydrogel" OR "silk scaffold" OR "silk composite" OR "silk bioink") AND ("nerve regeneration" OR "neural regeneration" OR neuroregeneration OR "nerve tissue engineering" OR neurite OR neurogenesis OR "neural stem cells"
